# Psychometric analysis of the Hip Disability and Osteoarthritis Outcome Score Joint Replacement (HOOS-JR)

**DOI:** 10.1016/j.ocarto.2024.100435

**Published:** 2024-01-11

**Authors:** Emilie N. Miley, Michael A. Pickering, Scott W. Cheatham, Lindsay Larkins, Adam C. Cady, Russell T. Baker

**Affiliations:** a Department of Orthopaedic Surgery and Sports Medicine, University of Florida, Gainesville, Florida 32607, USA; b Department of Movement Sciences, University of Idaho, Moscow, Idaho 83844, USA; c University of Idaho, Moscow, Idaho 83844, USA; d Kaiser Permanente, Woodland Hills, California 91367, USA; e WWAMI Medical Education Program, University of Idaho, Moscow, Idaho 83844, USA

**Keywords:** Total hip arthroplasty, Confirmatory factor analysis, Invariance testing, Patient-reported outcome measures

## Abstract

**Objective:**

Centers for Medicare and Medicaid Services (CMS) has requested hospitals collect and report patient-reported outcomes (PROs) beginning in 2024 including the Hip Disability and Osteoarthritis Outcome Score Joint Replacement (HOOS-JR). However, scale structural validity of the HOOS-JR has minimally been assessed. The purpose of this study was to assess internal consistency, structural validity, and multi-group invariance properties of the HOOS-JR in a large sample of patients who underwent a total hip arthroplasty (THA).

**Methods:**

A cross-sectional study using the Surgical Outcomes System was retrospectively queried for patients who underwent a THA. Internal consistency was assessed using Cronbach's alpha and McDonald's Omega. A confirmatory factor analysis (CFA) was performed on the HOOS-JR using *a priori* cut-off values. Multi-group invariance testing was also performed on the sample of patients across sex and age groups.

**Results:**

Internal consistency was acceptable for 6-item (alpha ​= ​0.88; omega ​= ​0.88) and 5-item (alpha ​= ​0.86; omega ​= ​0.86) HOOS-JR. The one-factor, 6-item CFA did not meet the recommended fit indices. The one-factor, 5-item CFA had acceptable fit for the sample data. Invariance testing criteria were met between the age groups; however, scalar invariance was not met for sex.

**Conclusion:**

The 6-item HOOS-JR did not meet contemporary model fit indices indicating that scale refinement is warranted. The 5-item met most goodness-of-fit indices and invariance criteria. However, further scale refinement may be warranted as localized fit issues were identified.

## 1 Introduction

Within orthopedics, total hip replacement (i.e., total hip arthroplasty [THA]) is one of the fastest growing surgical procedures performed each year [1,2]. Between 2000 and 2014, there was an estimated 132% growth in the number of primary THAs performed in the U.S and THAs are projected to increase by 71.2% by 2030 [3]. In addition, revision THAs are expected to grow between 43 and 70% by 2030 [2]. The fastest growing groups to undergo this procedure include females and those aged 45–84 years [4].

As the number of hip arthroplasties continue to rise, the Centers for Medicare and Medicaid Services (CMS) have requested hospitals collect patient-reported outcomes (PROs) beginning in 2024 [5]. Specific to patients undergoing THA, CMS has requested the collection of the Hip Disability and Osteoarthritis Outcome Score Joint Replacement (HOOS-JR) [5]. Unlike many PROs that currently exist to measure hip disability and pain, the HOOS-JR survey was developed as an alternative PRO to be shorter and less burdensome compared to the 40-item HOOS, yet specific for patients undergoing a THA [6].

The HOOS-JR was derived from the original HOOS, which consisted of 40 items in five domains: symptoms (S: five items); pain (P: 10 items); activity limitations-daily living (A: 17 items); sport and recreation (SP: four items); and hip-related QOL (Q: four items) [[6], [7], [8]]. The development of the HOOS-JR began with removal of the QOL domain (i.e., four items) because it did not address activities or movements specific to the hip [6]. Mean relevant scores were then calculated for each remaining HOOS item; items with a score of 66.6% or less were removed [6]. Redundant items (i.e., going up or down stairs, walking on a flat surface, standing upright, and walking on an uneven surface) were also removed from the item pool [6]. A principle component factor analysis (PCA) was then conducted on the remaining 30 items and a 30-item unidimensional solution was identified [6]. A Rasch analysis, which provides psychometric information about an instrument to facilitate logical and substantiated modifications, was performed on those items [9]. A final solution (i.e., the HOOS-JR) was accepted using only six items from the original 40-item: two items from the pain subscale and four items from the activity limitations-daily living subscale [9].

Minimal psychometric examination of the HOOS-JR has been conducted since its creation, with most of the work focused on the validity, reliability, and responsiveness of the instrument. The HOOS-JR has been reported to have acceptable internal consistency (0.86–0.87) and high responsiveness (0.80) [6]. Criterion validity, assessed using Spearman's Correlation Coefficient, has been reported to be acceptable with moderate to high correlations with the HOOS subscales (0.60–0.94) [6,10]. The HOOS-JR has also been considered reliable based on internal consistency values (Cronbach's alpha ​= ​0.86) [10]; values ranging from 0.70–0.89 have been recommended to establish sound consistency without item redundancy [11,12].

Although this initial work on the HOOS-JR provides some evidence for instrument reliability and validity, factorial validity and measurement invariance across subgroups have not sufficiently been established in the literature. Researchers have recently indicated the HOOS-JR met some, but not all, model fit recommendations (CFI ​= ​0.965; TLI ​= ​0.941; IFI ​= ​0.965; RMSEA ​= ​0.133) in a mostly healthy population (e.g., self-defined healthy [N ​= ​453], acute injury [N ​= ​13]), chronic injury [N ​= ​98]) who completed all 40 items of the HOOS [13]. Multi-group invariance testing on a small sub-sample of participants in this study who were diagnosed with hip osteoarthritis (OA) and/or previous history of a total hip replacement indicated the HOOS-JR may accurately capture valid group differences between those with hip injury and disability, and those who are healthy which provides initial support for scale validity [13]. However, the small sample sizes, utilization of healthy participants, and potential item response influence (i.e., original 40-item HOOS) necessitates further psychometric testing of the HOOS-JR.

Thus, there is a need to complete more robust psychometric analysis of the HOOS-JR in a larger, more diverse sample of patients who have undergone THA. Further analysis steps include conducting a confirmatory factor analysis (CFA) to test the hypothesized factor structure of the HOOS-JR in an appropriate patient population and to conduct multi-group invariance tests to determine if the scale is generalizable and unbiased towards different groups. An invariant model across subgroups ensures individuals from different groups are interpreting the survey items and meanings of the items similarly, regardless of the group classification (e.g., male or female). This confirms scores from the instrument truly correspond with the construct and are not due to group specific designations; instrument multi-group invariance is necessary to ensure the instrument can be used to compare hypothesized group differences [[14], [15], [16]]. Therefore, the primary purpose of this study was to assess model fit of the HOOS-JR in a large, diverse sample of patients who underwent a THA to examine its psychometric properties. The secondary purpose was to conduct multi-group invariance testing of the scale between age groups and sex on the parsimonious scale structure identified.

## 2 Methods

Surgical Outcomes System (SOS; Arthrex, Inc., Naples, FL) was retrospectively queried for the years 2014–2020 to establish a large data set to assess the psychometric properties of the HOOS-JR. The SOS is a securely maintained database that contains information submitted from surgical centers worldwide. The University Institutional Review Board (IRB) indicated approval for this study was not required because analysis of the de-identified data set from the SOS database was not considered human subject research. However, IRB approval was granted by the Cedar-Sinai Office of Research Compliance and Quality Improvement as part of a larger research project using SOS data. All patients who underwent a THA and completed the HOOS-JR were queried for responses. De-identified responses to the HOOS-JR and necessary demographic information (e.g., sex, ethnicity, race, age at treatment) utilized for multi-group invariance testing were downloaded from the SOS for analysis in this study.

### 2.1 Hip disability and osteoarthritis outcome scale joint replacement

Participants rated how frequently they engaged in the behaviors over the past week using a 5-point Likert scale (0 ​= ​none, 1 ​= ​mild, 2 ​= ​moderate, 3 ​= ​severe, and 4 ​= ​extreme) for all six items [6]. The responses to each item (i.e., raw score) are first summed together on a range from 0 to 24, with 0 indicating perfect patient-perceived hip health [6]. Raw scores may then be converted to an interval score ranging from 0 (raw score of 24) to 100 (raw score of 0) [6]. Raw item scores were used for the purpose of this study.

### 2.2 Data analysis

Data were exported from the SOS and downloaded using Microsoft® Excel for Mac (Version 16.46; Redmon, WA). Data were uploaded to Statistical Package for Social Sciences Version 28.0 (IBM Corp., Armonk, NY) for data cleaning and analysis. Missing data pertaining to the HOOS-JR were subject to the multiple imputation method; this process replaces the missing values with a set of random values based on the distribution of the sample [17,18]. Because the primary purpose was to assess the HOOS-JR, individuals with missing demographic data were not excluded from analysis and were left as missing values. Histograms, skewness, and kurtosis values were used to assess the normality of the data. Univariate outliers were assessed to determine if the z-scores exceeded the cut-off value of |3.3|. Multivariate outliers were also assessed using descriptive statistics and Mahalanobis distance of ≥16.81 [15]; these data were identified using a chi-square table with degrees of freedom and p-value of 0.01 [15,19]. This methodology generated the final data set used for the analysis.

### 2.3 Internal consistency

Internal consistency was assessed by calculating Cronbach's alpha (*α*) and McDonald's maximum likelihood omega (*ω*) for the 6- and 5-item unidimensional scale [12,14,15,20]. An acceptable range for Cronbach's alpha and McDonald's maximum likelihood ratio was set at ≥0.70 and ≤0.89 [11,20]. Values <0.70 indicate inadequate internal consistency, while values >0.89 indicate item redundancy [12,14,15,20].

### 2.4 Scale structure - confirmatory factor analysis

The final data set was used to conduct a CFA using Analysis of Moment Structures (AMOS) software (IBM Corp., Armonk, NY) on the 6-item HOOS-JR. As the HOOS-JR is scored as a one-factor model, the scale was defined as a one-factor, 6-item model [6]. Full Information Maximum Likelihood Estimation was used to generate the parameter estimates [15,16]. Model fit statistics included the likelihood ratio statistic (CMIN), Goodness of Fit Index (GFI), Comparative Fit Index (CFI), Tucker-Lewis Index (TLI), Bollen's Incremental Fit Index (IFI), Root Mean Square Error of Approximation (RMSEA), and Standardized Root Mean Square Residual (SRMR) [15,16,21]. Model fit was evaluated based on *a* priori values: GFI ≥0.95, CFI ≥0.95, TLI ≥0.95, RMSEA ≤0.06, IFI; ≥0.95, SRMR ≤0.08 [15,16,[21], [22], [23], [24], [25]]. Given the influence small *df* can have on the RMSEA (i.e., potential model misfit) [[21], [22], [23], [24]], the SRMR and CFI held greater weight in decisions regarding model fit testing. While the RMSEA value was elevated in both models, the value for the SRMR (i.e., 6-item ​= ​0.043; 5-item ​= ​0.024) met the recommended criterion (≤0.08) [24,25]. In addition to assessing the overall goodness of fit, the interpretability, size, and significance of the model's parameter estimates (i.e., factor variances, covariances, and indicator errors) were reviewed to identify any localized areas of strain [14].

### 2.5 Multi-group invariance testing

The full sample was then subjected to multi-group invariance testing where appropriate based on CFA results [14,15,26]. AMOS software was utilized to perform the analysis across sex (i.e., male, female) and age (i.e., ≤44, 45 to 54, 55 to 64; 65 to 74, ≥75) subgroups [14,26,27]. This was accomplished using a set of hierarchical procedures with an increasing level of constraint to determine if the respective items of the construct were stable and approximately equal across groups [14,15,26].

The model then underwent configural, metric, and scalar invariance testing [14,15,26]. First, the configural invariance test placed all groups in the same model to ensure the same factors have similar items across sub-groups. Secondly, the metric model then tested if factor loadings were equal across sub-groups [14,16]. If the model met metric invariance requirements, equal variances (i.e., group differences) between groups were then assessed [14,16]. Lastly, the scalar invariance test ensured that item intercepts were equal across groups, which indicated the means were not determined or altered by external factors [14,16]. If the model met scalar invariance requirements, equal mean models (i.e., score differences) were tested between groups [16].

Model fit was compared using the CFI difference test (CFI_DIFF_) and the chi-square difference test (χ^2^_DIFF_), with a *p*-value cut-off of 0.01 [22,26]. Given the sensitivity of the χ^2^_DIFF_ test to sample size [22], the CFI_DIFF_ test held greater weight in decisions regarding invariance testing model fit. If a model exceeded the χ^2^_DIFF_ test, but met the CFI_DIFF_ test, invariance testing continued.

## 3 Results

A total of 12,640 patient responses were exported from the SOS database and were included in the analysis. A total of 425 patients were identified as multivariate outliers (i.e., Mahalanobis ≥16.81) and were removed from the analysis. Of the patients identified as outliers, 240 identified as females and 136 identified as males. Those deleted represented each age group (i.e., ≤44, 45–54, 55–64, 65–74, ≥75) with a mean age of 65.89 ​± ​12.29 (standard deviation; SD). Upon examination of the skewness and kurtosis of the sample, no items had a non-normal distribution (i.e., <3.3). The final sample comprised of 12,215 patients with an age range from 16 to 90 years (mean age ​= ​63.39 ​± ​10.84 years; median age ​= ​64.0 years), with females accounting for 49.5% (n ​= ​6051) of the sample (Table 1). In the sample, the largest age group represented was 65–74 (31.7%) followed by the 55–64 group (31.2%) (Table 1).

**Table 1:** Demographic information of full sample.

	Full Sample (n ​= ​12,215)
**Sex (%)**	
Male	5428 (44.4)
Female	6051 (49.5)
Missing	736 (6.0)
**Age** ​± ​**standard deviation**	63.4 ​± ​10.8
**Age Group (%)**	
44 and younger	553 (4.5)
45–54	1742 (14.3)
55–64	3813 (31.2)
65–74	3878 (31.7)
75 and older	1739 (14.2)
Missing	490 (4.0)
**Ethnicity (%)**	
Hispanic	85 (0.7)
Not Hispanic	1682 (13.8)
Patient declines to answer	38 (0.3)
Missing	10,410 (85.2)

### 3.1 Internal consistency

Cronbach's alpha (*α*) and McDonald's maximum likelihood omega (*ω*) was conducted on the full sample of patients undergoing a THA at baseline (i.e., preoperative). Cronbach's alpha (*α*) and McDonald's omega (*ω*) were acceptable for the 6-item unidimensional scale (*α* ​= ​0.88; *ω* ​= ​0.88). In addition, Cronbach's alpha (*α*) and McDonald's omega (*ω*) were acceptable for the 5-item unidimensional scale (*α* ​= ​0.86; *ω* ​= ​0.86).

### 3.2 Scale structure - confirmatory factor analysis

**Fig. 1:**
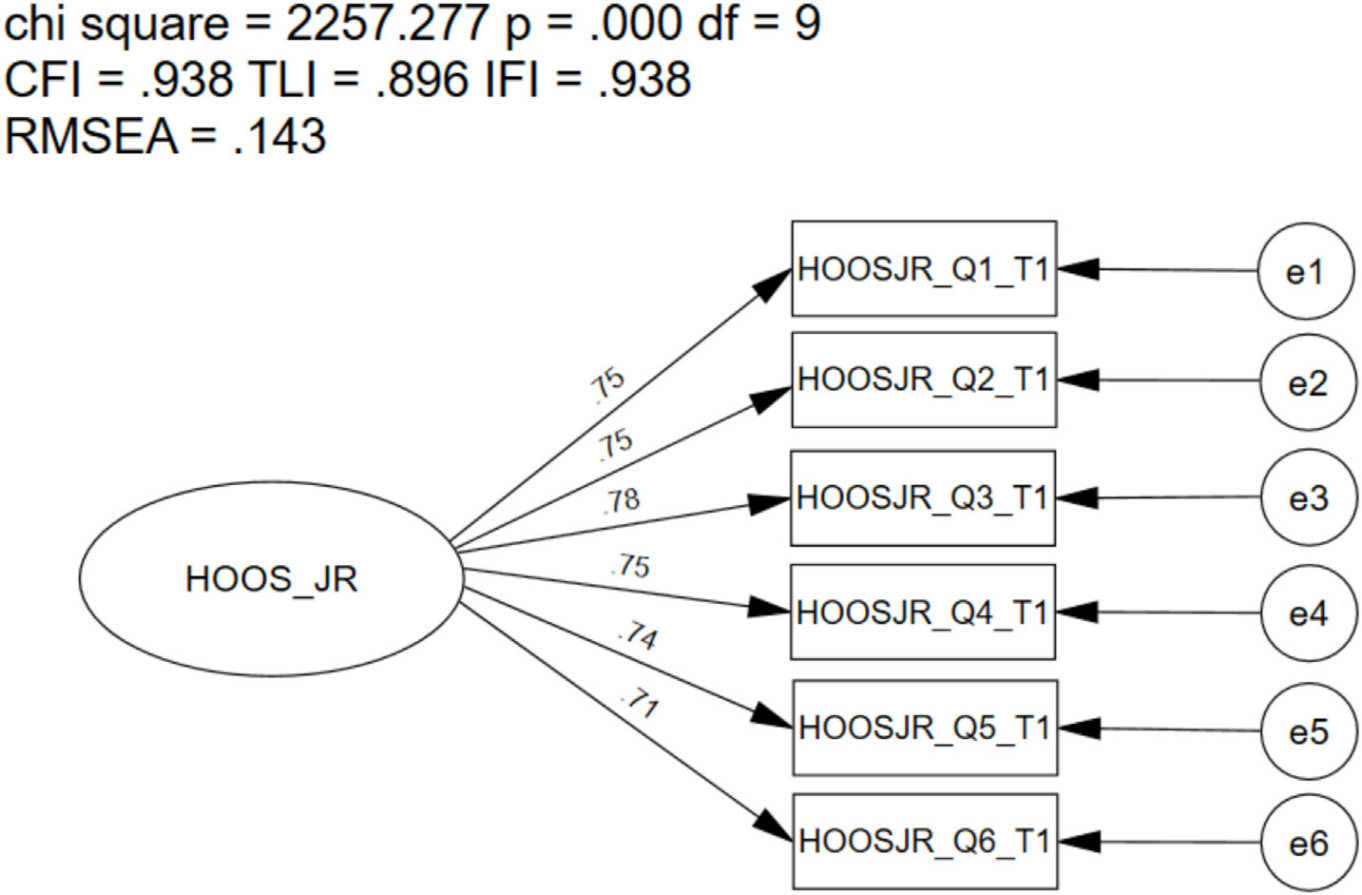
Confirmatory Factor Analysis of the One-Factor 6-item Hip Disability and Osteoarthritis Outcome Score for Joint Replacement (HOOS-JR). ChiSq, Chi Square (χ^2^); df, degrees of freedom, p, alpha level; CFI, Comparative Fit Index; TLI, Tucker-Lewis Index; IFI, Bollen's Incremental Fit Index; RMSEA, Root Mean Square Error of Approximation; SRMR, Standardized Root Mean Square Residual.

The one-factor, 6-item CFA of the HOOS-JR did not meet the recommended fit indices for the CFI (0.938), TLI (0.896), IFI (0.938), or RMSEA (0.143) (Fig. 1); SRMR did meet recommended levels (0.043). Factor loadings ranged from 0.71 to 0.78 and were significant (p ​≤ ​0.001). Modification indices revealed significant and meaningful cross-loadings between several items were present. Additionally, high error-term correlations were identified between items one and two (i.e., item one [going up or down stairs], item two [walking on an uneven surface]; 1476.92); also, between items five and six (i.e., item five [lying in bed turning over, maintaining hip position], item six [Sitting]; 584.39).

**Fig. 2:**
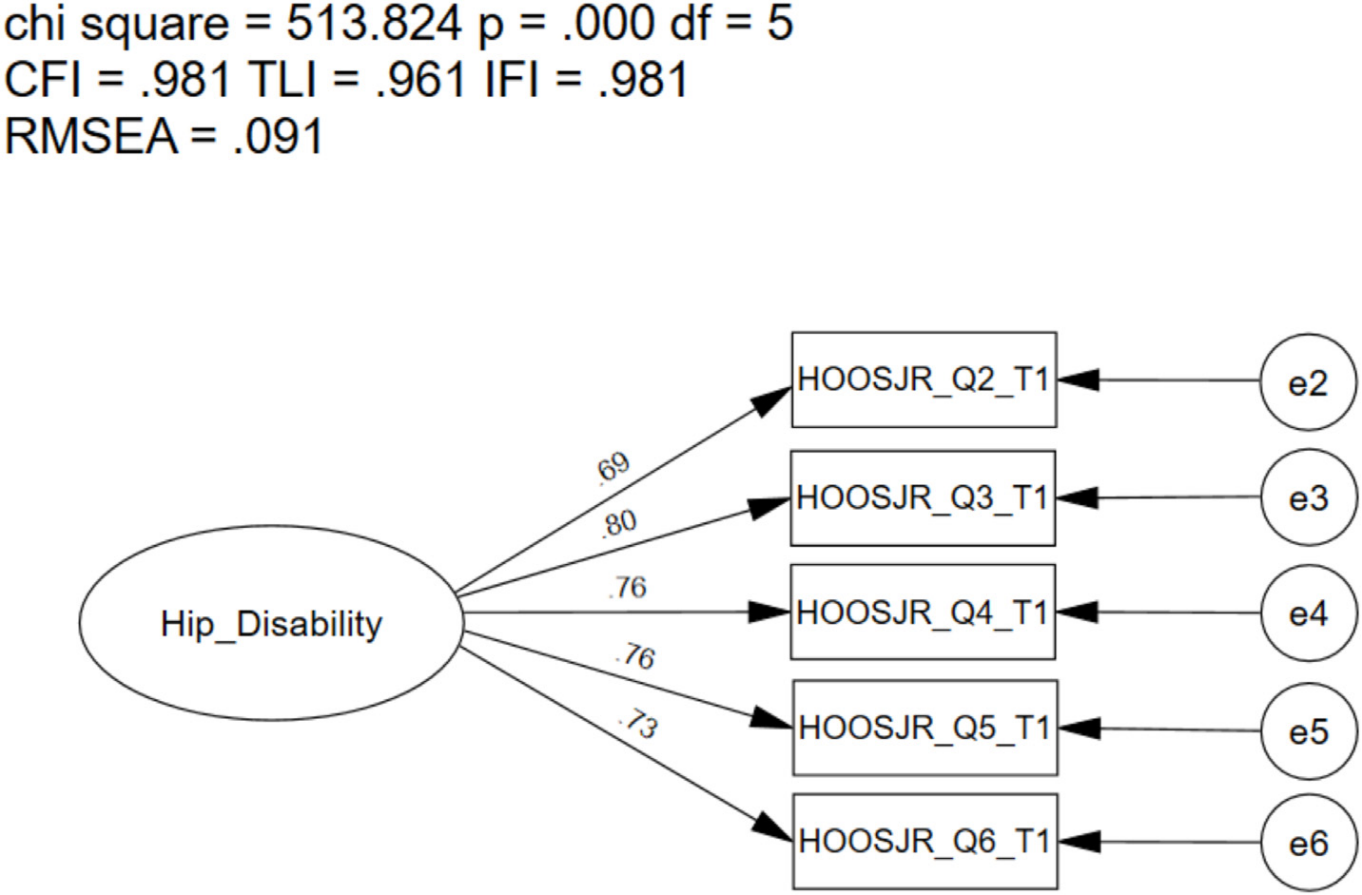
Confirmatory Factor Analysis of the One-Factor 5-item Alternate Hip Disability and Osteoarthritis Outcome Score for Joint Replacement (HOOS-JR) Model. ChiSq, Chi Square (χ^2^); df, degrees of freedom, p, alpha level; CFI, Comparative Fit Index; TLI, Tucker-Lewis Index; IFI, Bollen's Incremental Fit Index; RMSEA, Root Mean Square Error of Approximation; SRMR, Standardized Root Mean Square Residual.

As there was a high error-term correlation identified between items one and two, in addition to the lack of overall-fit, we explored the removal of item one. The one-factor 5-item CFA of the HOOS-JR met the recommended fit indices for the CFI (0.981), the TLI (0.961), and the IFI (0.981) (Fig. 2). The RSMEA value did not meet the recommended model fit guidelines (0.091); however, SRMR met the recommended model fit indices recommendations (0.024). Modification indices revealed significant and meaningful cross-loadings between several items were present. Additionally, there was a high error-term correlation present between items five and six (i.e., item five [lying in bed turning over, maintaining hip position], item six [Sitting]; 328.32).

### 3.3 Multi-group invariance testing

#### 3.3.1 Sex subgroups

Invariance testing was performed on the sample of patients who either identified as male or females (n ​= ​11,479), with missing data excluded from the analysis. The initial model (configural) demonstrated acceptable model fit (CFI ​= ​0.980; χ^2^ [4] ​= ​499.93; RMSEA ​= ​0.065; Table 2), indicating equal form across sex. The metric model (i.e., equal loadings) passed both the CFI_DIFF_ test and the χ^2^_DIFF_ test; as the model satisfied invariance criteria, the equal latent variance model was conducted. Both the CFI_DIFF_ test and the χ^2^_DIFF_ test both met invariance, indicating variances were equal across sex. The scalar model (i.e., equal loadings and intercepts) only slightly exceeded the CFI_DIFF_ test (i.e., 0.013); as such, we explored the latent means across sex. The equal latent means model did not meet model fit criteria; further assessment of the means indicated females reported significantly higher scores (i.e., more hip disability) than males.

**Table 2:** Goodness-of-fit indices for measurement invariance of the 5-item HOOS-JR across sex.

Modified 5-item HOOS-JR	χ^2^	df	χ^2^_diff_ (df_diff_)	CFI	CFI_diff_	TLI	RMSEA
Males (n ​= ​5428)	322.514	5	–	0.973	–	0.946	0.108
Females (n ​= ​6051)	173.696	5	–	0.987	–	0.974	0.075
Configural (equal form)	496.212	10	–	0.980	–	0.960	0.065
Metric (equal loadings)	499.927	14	3.715 (4)	0.980	<0.001	0.972	0.055
Equal factor variances^a^	500.960	15	4.748 (5)	0.980	<0.001	0.980	0.053
Scalar (equal indicator intercepts)	831.340	18	**335.128 (8)**	0.967	**0.013**	0.963	0.067
Equal latent means^a^	919.651	19	**423.439 (9)**	0.963	**0.017**	0.961	0.064

a Substantive questions; **Bolded** ​= ​did not meet cuff off criteria.

#### 3.3.2 Age subgroups

Invariance testing was performed on the sample of patients with a reported age (n ​= ​11,725); those with a missing age were excluded from the analysis. The initial model (configural) demonstrated acceptable model fit (CFI ​= ​0.980; χ^2^ [20] ​= ​522.74; RMSEA ​= ​0.041; Table 3), indicating equal form across age groups. The metric model (i.e., equal loadings) passed both the CFI_DIFF_ test and the χ^2^_DIFF_ test; as the model satisfied invariance criteria, the equal latent variance model was conducted. Both the CFI_DIFF_ test and the χ^2^_DIFF_ test both met invariance criteria, indicating variances were equal across age groups. The scalar model (i.e., equal loadings and intercepts) passed both the CFI_DIFF_ test and the χ^2^_DIFF_ tests; as such, the model allowed for comparison of the latent means across age groups. The equal latent means model did not meet model fit criteria; further assessment of the means indicated those who were in the age group of 55–64, 65–74, and >75 reported significantly lower scores (i.e., less hip disability) than those in the age groups <45 and 45–54.

**Table 3:** Goodness-of-fit indices for measurement invariance of the 5-item HOOS-JR across age.

Modified 5-item HOOS-JR	χ^2^	df	χ^2^_diff_ (df_diff_)	CFI	CFI_diff_	TLI	RMSEA
44 and younger (n ​= ​553)	21.005	5	–	0.973	–	0.987	0.076
45-54 (n ​= ​1742)	95.142	5	–	0.974	–	0.948	0.102
55-64 (n ​= ​3813)	193.170	5	–	0.978	–	0.955	0.099
65-74 (n ​= ​3878)	161.703	5	–	0.980	–	0.960	0.090
75 and older (n ​= ​1739)	54.728	5	–	0.988	–	0.975	0.076
Configural (equal form)	525.739	25	–	0.980	–	0.960	0.041
Metric (equal loadings)	540.622	44	14.883 (19)	0.980	<0.001	0.976	0.032
Equal factor variances^a^	559.587	45	33.848 (20)	0.979	0.001	0.977	0.031
Scalar (equal indicator intercepts)	694.998	57	169.259 (32)	0.974	0.006	0.978	0.031
Equal latent means^a^	847.713	61	**321.97 (36)**	0.968	**0.012**	0.974	0.033

a Substantive questions; **Bolded** ​= ​did not meet cuff off criteria.

## 4 Discussion

The purpose of this study was to assess the scale structure and multi-group invariance testing (i.e., sex and age groups) using CFA procedures of the 6-item HOOS-JR, as this outcome is commonly used in clinical practice following THA. Contemporary CFA procedures provide a more rigorous examination of the HOOS-JR for model fit and multi-group invariance. Overall, the 6-item model did not meet fit recommendations; however, the 5-item model met most contemporary fit recommendations. It may be prudent to alter the original 6-item HOOS-JR to ensure that a suitable tool exists to measure pain and functional disability following a THA.

### 4.1 Scale structure - confirmatory factor analysis

When defined as a one-factor, 6-item structure, the model did not meet the recommended overall goodness-of-fit (i.e., model fit indices) at baseline (i.e., preoperative). Model fit was poor (CFI ​= ​0.938; TLI ​= ​0.896; IFI ​= ​0.938; RMSEA ​= ​0.143; SRMR ​= ​0.043), with specific concerns related to item correlations and error-term covariances. The findings in this study are different than previous findings [13]; previous model fit criteria met some model fit indices (CFI ​= ​0.965; TLA ​= ​0.941; IFI ​= ​0.965) [13]. However, the previous study included a population sample of mostly healthy and younger individuals, whereas the present study includes a population sample whose mean age is higher and those undergoing a THA.

Modification indices revealed significant covariances between error terms (i.e., items one and two; items five and six). Moreover, upon further review of the items, item one (i.e., going up or down stairs) and item two (i.e., walking on an unstable surface) had a significantly high error-term correlation (1476.92) indicating overlap between the two items [15,16]; this finding is similar to previous literature [13]. Additionally, item one is considered double barreled, meaning that the item may be asking the patient to report about their pain performing more than one activity (i.e., going up or down stairs) [28].

Because item one was identified to have a high meaningful error-term covariance in multiple studies, and the item structure is considered double-barreled [28], we determined there was theoretical support for removal of this item. Upon removal, the defined one-factor, 5-item structure met most recommended model fit indices (CFI ​= ​0.981; TLI ​= ​0.961; IFI ​= ​0.981). Upon assessment of the modification indices, the high error-term covariance (328.32) was still identified between item five (i.e., lying in bed [turning over, maintaining hip position]) and item six (sitting).

While this analysis met most of the goodness-of-fit indices, the RMSEA was higher than the recommended best practices value (i.e., ≤0.06) in the 5-item (i.e., 0.091) model [14,15,24,25]; however, it has been recommended that RMSEA be interpreted with caution with small df models due to the potential for incorrectly rejecting a correctly specified model [21,24]. Recently, it has been recommended in the literature to report the SRMR instead of RMSEA in models with small df [21,24]. As such, we decided to report both the RMSEA and the SRMR given the small *df* in our model. While the RMSEA value was elevated in both models, the value for the SRMR (i.e., 6-item ​= ​0.04; 5-item ​= ​0.02) met the recommended criterion (≤0.08) [24,25].

Findings from these analyses indicate that modifications to the scale are warranted. As the HOOS-JR is required by CMS to be collected in patients undergoing a THA [29], a psychometrically sound instrument is needed for clinicians to accurately draw conclusions on outcomes within their clinical practice, and for CMS to have an instrument for accurate reimbursement [29,30]. Therefore, modifications to the HOOS-JR, such as rewording items, removing items, or creating new items, are necessary to create a PRO that effectively measures hip disability and pain [28].

As the HOOS-JR 6-item model did not meet recommended model fit criteria, further testing (i.e., multi-group invariance testing) is not recommended [[14], [15], [16]]. Additionally, it remains prudent to consider altering the instrument prior to its continued use to ensure clinicians are capturing appropriate surgical outcomes in a variety of patient populations. However, due to the frequent and necessary use of collecting the HOOS-JR in clinical practice for clinicians currently, we determined that there is a need to better understand the scale. As the 5-item model met most model fit indices, multi-group invariance testing of the one-factor 5-item model was performed.

### 4.2 Multi-group invariance testing

Invariance testing may be conducted for several reasons including, but not limited to, the assessment of an instrument's items to ensure similar interpretation across groups (e.g., age groups, sex), assessment of the underlying construct (e.g., hip disability) to ensure similar measurement across groups, or assessment of measurement properties across repeated measures (i.e., longitudinal) [15,16]. As such, multi-group invariance testing was conducted to determine if the HOOS-JR items were being interpreted similarly across subgroups (i.e., sex and age) and if the construct (i.e., hip disability) is being measured similarly across groups [15,16]. To our knowledge, this was the first study to perform multi-invariance procedures on the HOOS-JR in a pure sample of joint replacement patients. Additionally, given its heavy use in clinical practice, conducting invariance testing across multiple groups is pertinent to determine if the scale is adequately measuring patient's perceived hip disability across groups.

The initial (i.e., CFA) results indicated that the one-factor 5-item model met the stringent goodness-of-fit standards [14,15]; however, understanding the outcome in its current form (i.e., 6-item scale) is essential while further scale modifications occur. Concerns with the 5-item HOOS-JR were identified when multi-group invariance testing between sex was performed. Baseline models for males and females were performed; males met some model fit indices (CFI ​= ​0.97, TLI ​= ​0.95; IFI ​= ​0.97; RMSEA ​= ​0.11; SRMR ​= ​0.03), females met model fit indices (CFI ​= ​0.99; TLI ​= ​0.97; IFI ​= ​0.99; RMSEA ​= ​0.08; SRMR ​= ​0.02). The configural model (i.e., equal form) meet model fit recommendations, meaning group differences in variances were not noted in pain and function between sex. As the scalar model only slightly exceeded contemporary model fit recommendations, equal means model was explored. When the means were constrained to be equal, the model exceeded the recommended model fit indices; further assessment revealed that females report more pain and less function when compared to males. Due to the lack of scalar invariance, these findings indicate that males and females do not conceptualize hip disability similarly when using the 5-item HOOS-JR.

Multi-group invariance testing was also performed on the 5-item HOOS-JR across several age groups. Our results identified that the 5-item HOOS-JR is invariant across the examined age groups which indicated the scale can be used to assess age group differences in hip pain and function across these groups. Additionally, we found statistically significant latent mean differences between two specific age groups, with those aged 55–90 reported lower scores (i.e., less hip disability) than those aged 19–54. This is consistent with other authors who report two distinct patterns of activity levels (i.e., ≥55 and <55) pre-to postoperative, with those <55 reporting lower activity levels postoperatively when compared to the older group [31]. Additionally, several factors (e.g., level of activity, number of comorbidities, body mass index, American Society of Anesthesiologists score, medication use, etc.) have been identified in older populations (i.e., older than 55) that could explain the findings [31,32]. Specifically, these factors may contribute to older patients being less active which may then result in lower self-reported scores.

Even though our data represents a large sample of patients who underwent a THA, we were unable to obtain the associated diagnoses in this population. Therefore, it should be recognized that regardless of the diagnoses (e.g., OA, osteonecrosis, periprosthetic joint infection), comorbidities, medication use, etc., patients interpreted the questions similarly across the age groups and the means are not driven or contaminated by outside factors (e.g., cultural norms, group specific attributes). However, further research is needed to understand these age group differences on the outcomes pre- and post-surgical intervention.

### 4.3 Limitations and future research

Several limitations are present with our study. While a diverse population was assessed globally, most of the responses were collected from the United States (i.e., 12,197). Thus, future research should include psychometric assessment of the scale in samples across other countries as well to ensure appropriate measurement properties across cultures and languages. Additionally, upon export of the sample, we were unable to capture race and ethnicity due to a high amount of missing data (i.e., greater than 10,000 cases). As such, we were unable to assess the psychometrics of the scale across these groups within the sample. Future research should conduct invariance testing across race and ethnicity to ensure the scale has the necessary properties to support between groups analysis in these populations. Further, we were unable to determine if the HOOS-JR was collected across different medical history, and surgical (e.g., laterality, approach, primary vs. revision, inpatient vs. outpatient) or diagnostic (e.g., grade of OA, periprosthetic joint infection) variables due to the limitations of the database; thus, caution is warranted when examining HOOS-JR differences in groups that have not been analyzed. As this instrument is typically collected across time to assess outcomes following a THA, future research should also be conducted using similar methods to confirm the psychometric properties of the one-factor, 5-item HOOS-JR across time (e.g., longitudinal invariance testing) to ensure the scale can be used to assess change with repeated measures).

## 5 Conclusion

Our findings do not support the use of the one-factor, 6-item HOOS-JR and should be used with caution in clinical practice and research. However, invariance properties of the one-factor 5-item HOOS-JR meets most model fit indices and supports the use of the scale to assess differences between different age groups. Further scale modifications are warranted to develop a structurally sound PRO sensitive to measure change in patients undergoing a THA.

## Author Contributions

Dr. Emilie Miley confirms the following contributions to this study: study conception, design, statistical analyses, interpretation of results, and manuscript preparation. In addition, Dr. Russell Baker attributed to the following: reviewing the study conception, assisted in gaining access to data, obtaining institutional review board approval, reviewed methods and results, provided mentorship during statistical analyses, and contributed manuscript preparation. Dr. Scott Cheatham, Dr. Lindsay Larkins and Adam Cady contributed to manuscript preparation. Dr. Tony Pickering provided mentorship during statistical analyses and contributed to manuscript preparation. All authors reviewed the results and approved the final version of the following manuscripts.

## Funding

No funding was obtained for this study.

## Declaration of competing interest

The authors do not have any conflict of interest to disclose.
